# Treatment of adult attention-deficit hyperactivity disorder (ADHD) with transcranial direct current stimulation (tDCS): study protocol for a parallel, randomized, double-blinded, sham-controlled, multicenter trial (Stim-ADHD)

**DOI:** 10.1007/s00406-023-01652-4

**Published:** 2023-07-22

**Authors:** Nicole Mauche, Christine Ulke, Jue Huang, Annegret Franke, Holger Bogatsch, Thomas Ethofer, Oliver Grimm, Thomas Frodl, Knut Hoffmann, Georg Juckel, Sarah Kittel-Schneider, Aylin Mehren, Alexandra Philipsen, Christian Plewnia, Andreas Reif, Georg C. Ziegler, Maria Strauß

**Affiliations:** 1 Department of Psychiatry and Psychotherapy, Faculty of Medicine, University of Leipzig, Leipzig, Germany; 2 Clinical Trial Centre Leipzig, Faculty of Medicine, University of Leipzig, Leipzig, Germany; 3 Department of Psychiatry and Psychotherapy, LEAD Graduate School and Research Network, University Hospital of Tübingen, Tübingen, Germany; 4 Department of Biomedical Magnetic Resonance, University Hospital of Tübingen, Tübingen, Germany; 5 Department of Psychiatry, Psychotherapy and Psychosomatics, University Hospital Aachen, RWTH Aachen University, Aachen, Germany; 6 Department of Psychiatry, Psychotherapy and Preventive Medicine, Medicine Ruhr University, Bochum, Germany; 7 Department of Psychiatry, Psychotherapy and Psychosomatic Medicine, University Hospital, University of Wurzburg, Würzburg, Germany; 8 Department of Psychiatry and Psychotherapy, University of Bonn, Bonn, Germany; 9 Department of Psychiatry, Psychosomatic Medicine and Psychotherapy, University Hospital, Goethe University Frankfurt, Frankfurt am Main, Germany; 10 Department of Psychiatry and Psychotherapy, University Hospital, University of Leipzig Medical Center, University of Leipzig, Semmelweisstr. 10, 04103 Leipzig, Germany; 11 Department of Cognitive Neuroscience, Donders Institute for Brain, Cognition and Behaviour, Radboud University Nijmegen Medical Centre, Nijmegen, The Netherlands

**Keywords:** Adult ADHD, Transcranial direct current stimulation, Multicenter, Sham-controlled trial, Randomized, Brain stimulation

## Abstract

Transcranial direct current stimulation (tDCS) is a non-invasive brain stimulation treatment used as an alternative or complementary treatment for various neuropsychiatric disorders, and could be an alternative or add-on therapy to psychostimulants in attention-deficit hyperactivity disorder (ADHD). Previous studies provided some evidence for improvements in cognition and clinical symptoms in pediatric and adult ADHD patients. However, data from multi-center randomized controlled trials (RCTs) for this condition are lacking. Thus, our aim is to evaluate short- and mid-term effects of tDCS in this multi-center, randomized, double blind, and sham-controlled, parallel group clinical trial with a 1:1 randomization ratio. Primary endpoint is the total score of DSM-IV scale of the internationally established Conners’ Adult ADHD Rating Scales (German self-report screening version, CAARS-S-SR), at day 14 post-intervention (p.i.) to detect short-term lasting effects analyzed via analyses of covariance (ANCOVAs). In case of significant between-groups differences at day 14 p.i., hierarchically ordered hypotheses on mid-term lasting effects will be investigated by linear mixed models with visit (5 time points), treatment, treatment by visit interaction, and covariates as fixed categorical effects plus a patient-specific visit random effect, using an unstructured covariance structure to model the residual within-patient errors. Positive results of this clinical trial will expand the treatment options for adult ADHD patients with tDCS and provide an alternative or add-on therapy to psychostimulants with a low risk for side effects.

*Trial Registration* The trial was registered on July 29, 2022 in the German Clinical Trials Register (DRKS00028148).

## Background

Adult attention-deficit/hyperactivity disorder (ADHD) is a common neurodevelopmental disorder with a worldwide prevalence of at least 2.8% [1]. It is a childhood-onset disorder and characterized by the three-core symptoms attention-deficit, impulsivity, and hyperactivity [2]. In about 60% of pediatric patients, the symptoms persist into adulthood [3] and result in detrimental impacts on social, financial, and professional functioning [4]. The economic impact of adult ADHD places a significant burden on society. Despite the increasing awareness of ADHD, many affected adults are still underdiagnosed and untreated [5]. The overlap of ADHD symptoms with several other psychiatric disorders, including mood disorders, substance abuse, and anxiety, as well as the high incidence of comorbid psychiatric conditions is likely the reason for the high number of missed ADHD diagnoses in adults [1, 6]. The exact etiology of ADHD remains unclear. A multifactorial genesis with high genetic underpinnings [7] and imbalances in dopaminergic and noradrenergic systems is assumed [8].

Treatment guidelines for adult ADHD patients suggest multimodal therapy consisting of ADHD-specific medication and psychotherapy. Psychostimulants are recommended as the first-line medication for adults [9], but 30% of adult patients with ADHD do not respond to medication and its use may be limited by side effects and concerns of abuse [10, 11]. Non-medication treatments, have shown limited efficacy [12] and do not specifically target the underlying dysfunctional cortical activity [13]. Due to these limitations, there is an urgent need to evaluate and establish further treatment methods.

In addition to ADHD core Symptoms, ADHD patients also exhibit executive dysfunction in domains such as response inhibition or working memory [14], which are linked to the prefrontal cortex and its associated regions such as dorsolateral prefrontal cortex (DLPFC) [15]. Meta-analysis of functional Magnetic Resonance Imaging (fMRI) studies in ADHD showed consistent fronto-striato-parietal dysfunctions during tasks of inhibition and attention including the inferior frontal cortex, supplementary motor area, anterior cingulate cortex for inhibition, DLPFC, parietal, and cerebellar areas for attention [16]. Non-invasive brain stimulation treatments, such as transcranial direct current stimulation (tDCS), may be a suitable alternative treatment option, as they allow the targeted stimulation of functional altered key brain regions in ADHD, such as the prefrontal cortex and the fronto-subcortical system [16].tDCS applies a weak continuous electric current to the underlying brain via scalp electrodes with the electrical current passing between a positively charged anode and a negatively charged cathode. The transcranial application of weak direct currents to the human primary motor cortex is capable of eliciting intra-cortical excitability changes. The direction of these modulations depends on stimulation polarity: Anodal stimulation increases excitability, while cathodal stimulation decreases it. The respective changes evolve during the stimulation but remain for up to 1 h after the end of stimulation, given sufficiently long stimulation duration [17].

Until today, a small number of tDCS single-center studies have been conducted in adults with ADHD. A parallel, randomized, double blind, sham-controlled trial examined the efficacy of tDCS on the modulation of inhibitory control in adults with ADHD. Thirty patients were randomly allocated to each group and performed a go/no-go task before and after a single session of either anodal stimulation (1 mA) over the left DLPFC or sham stimulation. Data analysis showed no significant differences between the two groups regarding behavioral performance in the go/no-go tasks [18]. Another study applied double anodal stimulation of 1.8 mA tDCS for 20 min over the left and right DLPFC in 20 adult ADHD patients, which, compared to sham, improved only hyperactivity measures for a sustained attention task [19]. A double-blind crossover study applied three sessions of anodal 2 mA tDCS over the left DLPFC during working memory training in 37 adult ADHD patients. Compared to sham, anodal tDCS reduced commission errors in a sustained attention task immediately after treatment; however, the effect was gone three days after last stimulation [20].

A randomized, sham-controlled, double blind, crossover study showed that anodal tDCS over the left DLPFC modulated cognitive (reaction time) and physiological (P300 amplitude) measurements in the Eriksen flanker task in a state-dependent manner, but no effects were found in the stop signal reaction time of the stop signal task [21].

In a recent double-blind, randomized, sham-controlled crossover pilot study eleven pediatric ADHD patients underwent five consecutive sessions of cathodal 1.5 mA tDCS applied over the left DLPFC. Qualitative electroencephalography and participants behavioral responses were recorded. Compared to sham, immediately after the tDCS stimulation, alpha power increased in the right frontal area and delta power in the left frontal area while omission errors decreased, with no differences at follow-ups [22]. Finally, a meta-analysis of 14 tDCS studies, including 10 pediatric and 4 adult studies, reported limited evidence that 1 to 5 sessions, mostly of the DLPFC, improved clinical or cognitive measures of ADHD. The author’s summarize, a conclusive evidence from this meta-analysis is hampered by heterogeneity in stimulation protocols, sample age, and cognitive measurements. Furthermore, the authors call for larger, double-blind, randomized, controlled trials with homogeneous protocols testing both clinical and cognitive outcomes [23].

It is still unknown how long any improvement of ADHD symptoms lasts after applying tDCS. A few studies support the hypothesis of a lasting effect of tDCS with improved clinical symptoms beyond the end of stimulation. In children and adolescents, 20–30 min anodal stimulation over the left DLPFC for five days yielded superior results in the stimulation group, as compared to sham as assessed by neuropsychological [24, 25] and clinical measurements [24, 25]. The latter study describes lasting clinical effects, which were most prominent 7 days after the stimulation. Notably, there is evidence that tDCS application over multiple days increases the duration of its beneficial effects to several weeks [26–29], although these studies were not conducted in patients with ADHD. Examining persisting effects of tDCS on ADHD symptoms in adult patients, Cachoeira et al. [30] found strong effects (Cohen’s *d* > 1) in a pilot study (*N* = 17) concerning inattention, and moderate ones concerning hyperactivity/impulsivity two weeks post-intervention (p.i.). Four weeks p.i. the effect size in regard to inattention was moderate compared to sham stimulation, supporting the hypothesis of a lasting effect of tDCS with improved clinical symptoms beyond the end of stimulation. In their randomized controlled pilot study, 20 min anodal stimulation of the right DLPFC over five consecutive days was applied, with similar placement and time regimen in the sham group. In data obtained in our own working group we found further justification for active electrode placement on the right side [31]. In a [11C] MRB-PET/MRI study with adult ADHD patients, we found lower noradrenaline transporter availability in right than in left prefrontal-thalamic regions, which may indicate that prefrontal hypo-activation is more pronounced on the right side, justifying anodal tDCS stimulation on the right DLPFC.

Given some evidence of clinical and cognitive improvements with anodal tDCS over the right DLPFC, the main objective of this clinical trial is to systematically test the efficiency of anodal tDCS over the right DLPFC in reducing ADHD symptoms when used as an alternative or add-on therapy to stable ongoing treatment compared to sham stimulation. We hypothesize that the benefits will persist for at least two weeks after the end of the stimulation. Our stimulation protocol is based on the pilot study by Cachoiera et al. [30], which showed promising results in a small sample.

## Design and methods

This prospective study is designed as a multi-center, randomized, double blind, sham-controlled clinical trial and aims to demonstrate the superiority of anodal tDCS over the right DLPFC compared to sham stimulation in adult ADHD. Altogether, 250 patients will be randomly assigned (1:1 ratio) to the two parallel treatment arms. Patients will receive either experimental or control intervention. The experimental intervention consists of five 21-min sessions of bifrontal tDCS with the anode over right DLPFC (F4) and the cathode over the left DLPFC (F3) for five consecutive days over the course of one week. The control intervention consists of sham stimulation with identical electrode placement and identical timely regimen as in experimental intervention. Both interventions will be applied as alternative or add-on treatment to stable ongoing standard therapy. Study assessment with observer and self-ratings will be conducted for screening and baseline, every day during the treatment period and during the follow-up period at day 7, 14, 28, 56, and 90 p.i.

### Study population

The clinical trial will include in- and outpatients aged from 18 to 65 years with a primary diagnosis of adult ADHD according the DSM-5 criteria. Experienced study investigators will perform a clinical interview based on the Diagnostic and Statistical Manual of Mental Disorders 5th Edition (DSM-5 [32]). Table 1 contains all inclusion and exclusion criteria for the present study.

**Table 1:** Inclusion and exclusion criteria of the trial

Inclusion criteria	Primary diagnosis of ADHD according to DSM-5 diagnostic criteria
	Age between 18 and 65 years
	German speaking participants with ability to understanding informed consent
	Written informed consent
	ADHD-specific medication must be stable for at least 3 month before randomization
Exclusion criteria	Acute suicidality (based on personal assessment of the investigator and/or SCID-5-CV, item 9 marked as above threshold and/or BDI-II, item 9 > 2
	Acute severe depression episode (defined as ≥ 7 symptoms of MDD with a minimum of 3 main criteria
	Diagnosis of the following psychiatric disorders as primary clinical presentation:
	a. Persistent depressive disorder
	b. Psychotic symptoms
	c. Bipolar disorder
	d. Schizoaffective disorder
	e. Schizophrenia
	f. Psychosis
	g. Borderline personality disorder
	Diagnosis of current alcohol or substance use disorder (except for tobacco) as primary clinical presentation and/or a positive urine drug screening
	Change in ADHD-specific medication/s planned before assessment of the primary endpoint
	Current use/washing out of psychotropic medication (e.g., antidepressants, antipsychotics, anticonvulsants, lithium)
	Severe somatic comorbidities
	Severe neurological comorbidities (e.g., history of brain surgery, significant brain malformation or neoplasm, head injury, stroke, epilepsy, neurodegenerative disorder)
	Contraindications for tDCS intervention (e.g., mental plates or electronic implants in the brain or skull, skull defects and skin lesions on the scalp, history of epileptic seizure, cardiac pacemaker or defibrillator)
	Pregnant or nursing females

### Study centers and recruitment

Besides the Department of Psychiatry and Psychotherapy of the University of Leipzig (coordinating center), six additional psychiatric departments in Germany will participate in the trial. These centers must have the appropriate technical equipment and experienced staff regarding diagnosis and treatment of adult ADHD patients and in applying of tDCS in psychiatric conditions. During the pre-screening period, a trained study investigator will give comprehensive verbal and written information about trial-related objectives, procedures, and possible risks to each patient. All patients will have the opportunity to ask questions and will have enough time to consider whether they want to participate in the study. Before any study-specific measures are administered, an informed consent form must be signed. The consent to participate in the trial can be withdrawn at any given time during the study and without the necessity to provide a reason for withdrawal.

Continuous site monitoring will ensure the early identification of recruitment or performance problems. In case of relevant delays, site-specific measures will be taken, e.g., additional dedicated staff, alternative organizational structures, intensified social media activity, and further involvement of local patient groups. However, in case of persistent and substantial recruitment delays, the study center will be closed and a suitable new center will be identified and initiated.

### Patient involvement

To empower affected individuals and give patients a voice concerning their key concerns and symptoms to be included to evaluate subjective improvement in addition to clinical improvement, we closely cooperate with patients’ organizations (e.g., “Selbsthilfegruppe ADHS im Erwachsenenalter Leipzig”). In addition, we cooperated with our current outpatients affected by adult ADHD and their relatives during various stages of the preparation of the trial. As part of the trial-planning phase, we organized a meeting at the ADHD outpatient center of Leipzig University with members of local self-help groups and current outpatients to ensure that the overall goal and outcome of research is relevant to them. Further, we informed them about the planned study design and asked for their opinion about the selected methods, the frequency of visits and the acceptance of the tDCS devices. Additionally, we asked the participants about their opinions regarding the relevance of selected outcome variables for patients. Based on their feedback, we chose outcomes in accordance with patients’ experiences and revised the list of questionnaires to be included. Patient involvement throughout the whole duration of trial is planned. We will organize further meetings, in Leipzig, and if possible at the other centers, to ensure that the recruitment process is practical and feasible.

### Study timeline

**Fig. 1:**
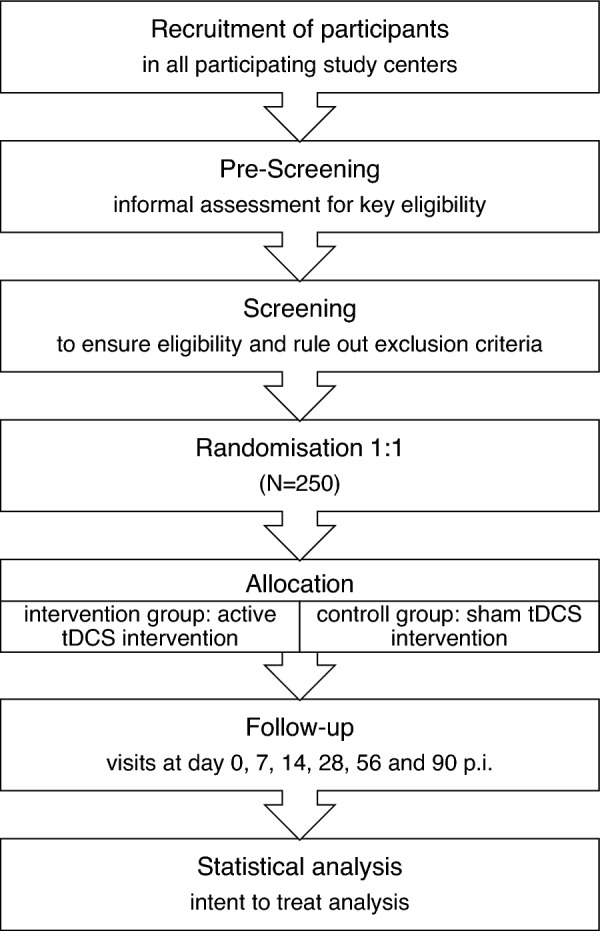
Study timeline

The trial comprises a pre-treatment phase, a treatment phase of 1-week duration, and a follow-up phase of 3 months. The flow chart (Fig. 1) gives an overview of the study timeline. In the context of a pre-screening, the most important inclusion and exclusion criteria are informally checked. If pre-screening examination confirms a suspected adult ADHD diagnosis, an appointment for screening will be offered to the patient concerned. Patients will receive the study information and consent in advance, allowing sufficient time for the consent form to be studied before it is being signed after a final discussion with the investigator. After informed consent, the screening takes place within 10 days prior to randomization and start of the intervention. It includes verification of the ADHD diagnosis as part of the review of the inclusion/exclusion criteria. Furthermore, severe psychiatric, somatic and neurological concomitant diseases will be excluded. Patients who meet the inclusion and exclusion criteria will complete all pre-treatment assessments comprising the baseline visit and will then be randomized to active or sham tDCS following the randomization procedure. During the treatment phase, each patient will receive one active/sham tDCS session per day on five consecutive days over the course of one week. At each tDCS session, possible adverse events will be checked. Major protocol violations such as missing one (or more) intended stimulations or an overall duration of treatment longer than 8 days will result in exclusion from per-protocol analysis.

During the follow-up phase, five visits are scheduled at day 7, 14, 28, 56, and 90 after the end of treatment. Patients’ participation in the trial will end with the last follow-up visit.

## Assessment

### Clinical measures

The assessments and the procedures performed during the pre-screening, screening, baseline, treatment period and follow-up visits are summarized in Table 2.

**Table 2:** Schedule of assessments and procedures

Study period	Pre-treatment		Treatment					Follow-up				
Visit	PS	SV	1	2	3	4	5	6	7	8	9	10
Week	−1		1					2	3	5	9	14
Day^a^		−10	1	2	3	4	5	12±2	19±2	33±4	61±4	95±7
*Assessments*			*Screening and consent*									
Informed consent for pre-screening	•											
ASRS-v1.1	•											
WURS-K	•	•										
Informed consent for clinical trial		•										
In-/exclusion criteria		•										
SCID-5-CV		•										
SCID-5-SPQ^b^		•										
SCID-5-PD^c^		•										
AUDIT		•										
DUDIT		•										
WST		•										
Functional level rating scale		•										
Physical and neurological exam		•										
Medical and psychiatric history		•										
Treatment status		•										
Concomitant medication			•^d^									
Urine drug test			•^d^									
Urine pregnancy test			•^f^									
Randomization			•^d^									

			*Intervention*									
Active/sham tDCS			•	•	•	•	•					

			*Efficacy*									
CAARS-S-SR		•	•^d^				•^e^	•	•	•	•	•
CPT			•^d^				•^e^		•			•

			*Safety*									
tDCS safety screening		•										
BDI-II		•	•^d^				•^e^	•	•	•	•	•
CRQ			•^e^	•	•	•	•^e^					
(S)AE (only during active/sham tDCS)			•^e^	•	•	•	•^e^	•				

			*Social functioning and quality of life*									
AAQoL			•^d^				•^e^	•	•	•	•	•
SCL-90-S			•^d^				•^e^	•	•	•	•	•
PSQI			•^d^				•^e^	•	•	•	•	•

			*Other*									
Demographics		•										
CGI-S			•^d^				•^e^	•	•	•	•	•
CGI-I							•^e^	•	•	•	•	•
Blinding check			•^e^				•^e^					

*PS* pre-screening, *SV* screening visit

^a^Related to randomization

^b^Screening questions for BPD only

^c^Inquiring of the positively screened items from the BPD section only

^d^Before active/sham tDCS

^e^After active/sham tDCS

^f^For females of child-bearing potential

In the context of the pre-screening, the German Adult ADHD self-report scale (ASRS-vI.I [33]) symptom checklist, for evaluation of current symptoms, and the short form of German Wender Utah Rating Scale (WURS-K [34]), for retrospective assessment of symptoms in a participant’s childhood, is filled out online by those interested in participating.

The screening visit includes the registration of the sociodemographic characteristics, the collection of the medical and psychiatric history, a physical–neurological examination, the documentation of treatment status and the tDCS safety screening. In addition, a detailed psychopathological examination is carried out with the clinician version of the Structured Clinical Interview for DSM-5® Disorders-Clinical Version (SCID-5-CV [35]), the Screening Personality Questionnaire (SCID-5-SPQ [36]) and the Section for Bipolar Personality Disorder (BPD) for Personality Disorders (SCID-5-PD [36]). In addition, the participants fill out the following questionnaires: Conners' Adult ADHD Rating Scales self-report screening form (CAARS-S-SR [37]), WURS-K, Beck Depression Inventory-II (BDI-II [38]), Alcohol Use Disorders Identification Test (AUDIT [39]), Drug Use Disorders Identification Test (DUDIT [40]), Wortschatztest (vocabulary-based IQ screening, WST [41]), and a functional level rating scale.

At the baseline visit and before the first active/sham tDCS, the following procedures and questionnaires will be performed: urine drug test, and if applicable, a urine pregnancy test, documentation of the concomitant medication, Adult ADHD Quality of Life Questionnaire (AAQoL [42]), CAARS-S-SR, BDI-II, Clinical Global Impression of Severity (CGI-S [43]), Continuous Performance Test (CPT [44]), Symptom Checklist‐90‐Standard (SCL-90-S [45]), and the Pittsburgh Sleep Quality Index (PSQI [46]).

At each active/sham tDCS session, every participant will be asked about adverse events and the Comfort Rating Questionnaire (CRQ [47]) will be performed. Additionally, all participants will be asked whether they believe they received active or sham stimulation after the first and last treatment session at visits 1 and 5 (“blinding check”).

The following procedures and questionnaires will be performed immediately after the last active/sham tDCS: documentation of any changes of the concomitant treatment, AAQoL, BDI-II, CAARS-S-SR, CPT, CGI-S, Clinical Global Impression of Improvement (CGI-I [43]), SCL-90-S, and PSQI.

Finally, during the follow-up period, the following assessments will be repeated on day 7 ± 2, 14 ± 2, 28 ± 4, 56 ± 4, and 90 ± 7 after end of intervention: documentation of any changes of the concomitant medication, AAQoL, BDI-II, CAARS-S-SR, CGI-S, CGI-I, SCL-90-S, and PSQI. CPT will be repeated at 14 and 90 days after the end of intervention.

### Randomization and blinding

All study participants will be randomized by the trial site using a secure online procedure provided by the ZKS Leipzig, which results in an automatic e-mail confirmation on successful randomization to the site itself and the ZKS Leipzig. Stratification by ADHD subtypes (combined, inattentive or hyperactive/ impulsive) and regular ADHD-specific medication/s at baseline (yes/no) was applied.

Randomization will take place as follows: The neuroConn GmbH generated the intervention code lists for active/sham tDCS procedure. The technical data required for stimulation (active/sham procedure) are uploaded in a cloud (Microsoft Azure Cloud) by neuroConn GmbH. The ZKS Leipzig receives intervention code lists for active/sham tDCS procedure from the neuroConn GmbH. The ZKS Leipzig transferred these intervention code lists into the randomization tool. During randomization, the result of the randomization is assigned to the patient-ID in a blinded manner. The trial site will download the technical data required for stimulation from the cloud using the intervention code. After end of intervention, the associated technical data of the stimulation performed will be uploaded to the cloud using the intervention code. The neuroCare group AG is able to monitor the uploaded data of the stimulations, so that problems or possible technical faults can be detected. Only technical data are transmitted via cloud, patient data are gathered outside the cloud. Thus, all sites remain blinded for the entire duration of the trial.

## Intervention

### Stimulation

Intervention will be conducted as five 21-min stimulation sessions on five consecutive days over the course of one week (5 sessions in total).

The 21-min stimulation will be administered with a bipolar DC-STIMULATOR MOBILE (neuroConn GmbH), using two 5 × 7 cm electrodes (inserted in saline-soaked sponges) with the anode over the right DLPFC (F4), cathode over the left DLPFC (F3); electrodes will be placed based on the international 10–20 system and fixed with a headband.

A constant current of 2 mA (current density = 0.0571 mA/cm^2^) will be applied in verum tDCS. Current will be ramped up/down for 30 s at the beginning/end of the session to avoid fast transients enabling subjects to distinguish between real and sham stimulations [48]. Output current, output voltage, and impedance are monitored continuously. If threshold values are exceeded, the DC-STIMULATOR MOBILE is switched off for safety reasons. The current stimulation is stopped in Safe-Stop mode. The Save-Stop mode prevents an uncomfortable and sometimes even painful “current jump” by slowing down the present current to 0 mA when stimulation is manual or automatic stopped.

### Sham stimulation

In contrast to the active tDCS condition, current for the sham intervention will be ramped up to 2 mA for 30 s followed by a 30 s fade out at the beginning and end of each session to mimic the experience of mild itching and tingling that is commonly reported during active stimulation [30], but no effective stimulation will occur for the remaining duration of the intervention. The induced sensory impression of being stimulated serves to improve the blinding.

### Technical devices

For active and sham tDCS, a neuroConn DC-STIMULATOR MOBILE with study mode is used (neuroConn GmbH, Ilmenau, Germany). This device is a microprocessor-controlled, battery-driven constant current source, complying with the Medical Device Directive of the European Union (CE-certified). The electrodes are separately inserted in 35 cm^2^ saline-soaked sponges before placed over the scalp. An elastic strap made of non-conducting material will be used to fix the electrodes in place. Application time, current range, and frequencies are programmable, settings can be saved. Active or sham stimulation mode is chosen by entering of different number codes.

### Blinding

The control arm receives sham stimulations that will be applied in a way that is indistinguishable from active stimulation, i.e., in the same time-schedule of intervention and at identical localizations.

For all data transfers between the trial site and the cloud, the EDSM—Energy and Data Storage Module is used as an integral component of the neuroConn DC-STIMULATOR MOBILE. The data transmitted via cloud, e.g., the technical data required for stimulation, cannot be changed by trial site. Thus, the blinding is guaranteed.

### Concomitant treatment

All medications that are approved according the guidelines for the treatment of adult ADHD in Germany are permitted as additional treatment during study participation. Other psychotropic medication is not allowed (i.e., benzodiazepines, z-hypnotics, antidepressants, neuroleptics). If present, the pharmacological therapy should be stable within the last 3 months before randomization and should be kept constant until assessment of the primary endpoint. Critical changes within the treatment period lead to exclusion from per-protocol analysis. Changes in medication and non-pharmacological treatments are continuously registered.

## Study endpoints

### Primary outcome measure

Primary objective is to test the hypothesis whether or not tDCS is effective in reducing ADHD symptoms with a benefit for at least 2 weeks after the end of stimulation and superior to sham stimulation determined by DSM-IV ADHD symptoms total score (DSM-ADHS) of the CAARS-S-SR. Potential lasting effects of active and sham stimulation will be monitored for 90 days p.i.

### Secondary outcome measures

As secondary endpoints, the following scores and safety assessments will be compared between active and sham stimulation:

- CAARS-S-SR DSM-IV scales: inattentive symptoms (DSM-IN) and hyperactive-impulsive symptoms (DSM-HY/I) on visit 1, 5, and all follow-up visits (6–10)
- Assessment of Reaction time, variability, omission, and action errors by CPT on visit 1, 5, 7, and 10
- ADHD-specific quality of life assessed by AAQoL on visit 1, 5, and all follow-up visits
- Symptomatic distress assessed by SCL-90-S on visit 1, 5, and all follow-up visits
- Sleep quality and disturbance assessed by PSQI on visit 1, 5, and all follow-up visits
- BDI-II before first and after last tDCS application and on all follow-up visits CRQ after each tDCS application
- Incidences of adverse clinically relevant findings

### Sample size calculation

The estimation of the requested sample size in Stim-ADHD was based on various data from our own former, in parts unpublished, research activities and studies. According to our pre-/post-data, moderate to high correlations between baseline and post-treatment data of *r* ≈ 0.39… > 0.7 were observed in previous populations, with lower values in pharmacotherapy, and *r* = 0.57 in the aggregated dataset. Because of the rather small samples and since no valid assumption can be made on the impact of the further stratification criteria on pre-/post-correlation, we conservatively chose *r* ≈ 0.5 for calculation of sample size.

PASS sample size software (http://www.ncss.com/software/pass/pass-documentation; version 14, 2016) was used, based on an ANCOVA design with baseline CAARS DSM-IV and the stratification criteria as covariates, means as reported and *R*^2^ = 0.25.

With a significance level of *α* = 5%, a randomization ratio 1:1, and 208 patients in total, a power of 81% will be reached to detect between-group differences of 3.5 points (assuming a small sham effect and/or regression to the mean in our control group as well as to be conservative in our assumptions).

The effect size of this design is determined by *f* = SD_group means_/SD SD_CAARS DSM-IV ADHD total post_ = 0.172. According to Cohen [49], *f* < 0.1 is regarded as small, *f *≈ 0.25 as moderate. With less conservative (but reasoned) assumptions on higher *R*^2^ (see observed pre–post-correlation) and lower SD_post_ (due to a rather homogeneous population and standardized intervention), and with possibly lower dropouts (because of the non-pharmacological intervention as seen in our patients), the power would increase and possibly smaller effect sizes may be detectable.

### Statistical analysis

The full analysis set [FAS, based on the intention-to-treat (ITT) strategy] contains all randomized patients with informed consent and (at least) a single study intervention performed. The per-protocol (PPS) comprises all patients belonging to the FAS without major protocol violations. To identify between-groups differences for the primary endpoint, an ANCOVA with treatment (tDCS vs. sham stimulation) as main factor and CAARS DSM-IV ADHD total at randomization, ADHD sub-type and pre-medication (stratification criteria) as covariates within the FAS will be performed.

If a significant between-groups difference is identified in the primary analysis, a linear mixed model (LMM) for repeated measurements will be applied to analyze the course of CAARS DSM-IV ADHD total, including 5 assessments per patient: at baseline, at days 14, 28, 56, and 90 post-intervention. For sensitivity analysis, multiple missing value (MV) imputations may be performed to support the results of the repeated measurements LMM and investigate the influence of MVs. No α adjustment for multiple testing is required. (Quasi-)metric secondary and safety endpoints will be analyzed analogously to the primary endpoint.

### Documentation, monitoring, and data management

All clinical data entered by the site staff into eCRFs will be recorded in a pseudonymized form exclusively using the patient’s identification code.

The Clinical Trial Centre Leipzig (ZKS Leipzig) will be responsible for trial monitoring. Pre-study, initiation, interim, and close-out visits will be performed in all centers. During the visits, the monitor will: (a) check informed consent forms of all patients enrolled, (b) perform targeted source data verification for patients with possible deviations, (c) perform source data verification of the key data in a random sample of at least 40% of the site’s patients (d) discuss open queries raised by data management or safety personnel check and update the investigator site file.

For creation of the trial database, the EDC tool secuTrial®, developed and distributed by interActive Systems GmbH (iAS), will be used. The information entered into the eCRF by an authorized member of the trial team will be systematically checked for completeness, consistency, and plausibility. The site staff is responsible for data correction and can resolve queries directly in the eCRF-page. During the whole course of the trial, daily backups of the data are made. Unauthorized access to patient data is prevented by the access concept of the trial database, which is based on a strict hierarchy and role concept. Any change of data is recorded automatically via audit trail within the database. At the end of the trial, once the database has been declared complete and accurate, the database will be locked.

### Safety aspects and data safety monitoring board

The exclusion criteria of this trial include all contraindications for the application of tDCS (e.g., metal plates or electronic implants in the brain or skull, skull defects, history of epileptic seizures, cardiac pacemaker). In addition, major depressive disorder (MDD) severity and suicide risk will be measured during treatment and follow-up visits with the BDI-II due to the theoretical risk of worsening or new development of depressive symptoms. This risk arises as the electrode placement in our study is opposite to the usual placement in depression treatment.

Information on adverse events (AEs) and serious adverse events (SAEs) will be collected during treatment period and up to the first follow-up visit after treatment period. (S)AE will be followed up until complete recovery and/or patient’s status is stable. A Data Monitoring Committee (DMC), consisting of three independent experts without conflicts of interest, will meet periodically to perform a review of the accumulated study data regarding the safety of the trial intervention as well as the integrity and validity of the data.

## Discussion

Here, we report on the design and rationale of a clinical trial conceived to investigate the efficacy of anodal tDCS over the right DLPFC in reducing ADHD symptoms as an alternative or add-on therapy to stable ongoing treatment compared to sham stimulation. There is a lot of evidence that tDCS can lead to an improvement in ADHD symptoms; however, it is still unclear how it can be used in routine clinical practice. To our knowledge, this study represents the first attempt to investigate the effect of tDCS on ADHD symptoms in adults systematically in a multi-center study design.

Over last years, several small tDCS studies with heterogeneous study designs have been conducted in patients with ADHD. The majority of these studies have been conducted in pediatric ADHD patients, presumably due to the high tolerability and relatively low side effect profile of tDCS [50]. Based on the dysfunction findings in fMRI studies conducted in ADHD over the last two decades [16], most of them used tDCS in either one or five sessions targeting mostly DLPFC. Meta-analyses of tDCS effects, mostly over DLPFC, show small effect sizes for improved cognition [23, 51]. However, only a small number of studies have measured clinical improvement in ADHD patients using tDCS, with inconsistent findings [50]. The authors of a meta-analysis of 10 pediatric and 4 adult tDCS studies summarize that a conclusive result is hampered by heterogeneity in stimulation protocols, sample age, and cognitive measures. Therefore, the authors call for larger, double-blind, randomized, controlled trials with homogeneous protocols testing both clinical and cognitive outcomes in ADHD [23]. Almost all authors of reviews and meta-analyses on the use of tDCS in ADHD also conclude that the efficacy of tDCS, and especially its clinical benefit on ADHD symptoms, cannot yet be conclusively assessed and further studies with optimized designs are warranted (see as example [52]). With our study, we aim to contribute to gathering more robust data on this.

The intention of this clinical trial is to include both medicated and unmedicated ADHD patients, to investigate the effects of tDCS as an add-on therapy to the treatment adhering to standard ADHD guidelines as well. Thus, the design is primarily intended to reflect clinical reality, because in our opinion the investigation of tDCS as a stand-alone therapy would only be applicable to a small group of clinical patients. Another important point in the preparation of our study was the choice of primary and secondary endpoints. In order to choose measures that are also relevant from a patient perspective, we involved patients and their relatives in this decision. Previous studies mostly investigated either the effects of tDCS on neuropsychological symptoms or clinical symptoms [52], however we decided to survey both aspects, including quality of life parameters in our study, which was also supported by the patients.

Concerning electrode placement, stimulation intensity, and frequency, the methodological decision was based on the above mentioned evidence. The lateralization of the excitatory electrode over right DLPFC was supported by neuroimaging study of our own working group, which indicated that prefrontal hypo-activation is more pronounced on the right side [31]. The selection of the stimulation parameters was based on the previous pilot study by Cachoiera et al. [30], which showed significant lower self-reported ADHD symptoms after active tDCS in comparison with sham stimulation. In order to measure short-term and also possible mid-term effects, we significantly extended the follow-up period with visits up to day 90 after the intervention. Potentially observed long-lasting effects would be instrumental in clinical implementation of tDCS in ADHD.

In summary, positive results of this parallel, randomized, double-blinded, sham-controlled, multi-center trial would expand the treatment options for adult ADHD patients with an alternative or add-on therapy to psychostimulants with a low risk for side effects. Detailed reporting of study protocols is intended to increase transparency in clinical research.

### Trial status

Enrollment for the study began in September 2022. At the time of submission, we have enrolled 28 participants at two study sites (Leipzig, Wurzburg).
